# Hypermentalizing as a marker of borderline personality disorder in Italian adolescents: a cross-cultural replication of Sharp and colleagues’ (2011) findings

**DOI:** 10.1186/s40479-019-0104-5

**Published:** 2019-04-10

**Authors:** Antonella Somma, Mauro Ferrara, Arianna Terrinoni, Claudia Frau, Ignazio Ardizzone, Carla Sharp, Andrea Fossati

**Affiliations:** 1 Faculty of Psychology, Vita-Salute San Raffaele University, via Stamira d’Ancona 20, 20127 Milan, Italy; 2 Department of Human Neurosciences, Sapienza University of Rome, Via dei Sabelli, 108, 00185 Rome, Italy; 3 Health and Biomedical Sciences Building, The University of Houston, 4811 Calhoun Rd. - Rm 373, Houston, TX 77204 USA

**Keywords:** Borderline personality disorder, Mentalization, Adolescence, Movie for the assessment of social cognition, Inpatients

## Abstract

**Background:**

Extant literature indicates that Borderline Personality Disorder (BPD) may be reliably assessed in adolescence. Sharp and colleagues’ (2011) suggested that mentalization could be an important early target for intervention in BPD adolescents and showed that hypermentalizing may represent an important marker to distinguish emerging BPD from adolescent turmoil. We aimed at testing if both dimensionally-assessed and categorically-diagnosed BPD was selectively associated with hypermentalizing errors on the Movie for the Assessment of Social Cognition (MASC) task in Italian adolescent inpatients and community adolescents.

**Findings:**

The sample was composed of 58 Italian adolescents who were consecutively admitted to an adolescent psychiatry unit in Rome, Italy. BPD was assessed using the Structured Clinical Interview for *DSM-5* Personality Disorders (SCID-5-PD); the MASC task was used to assess mentalizing. Findings supported the hypothesis of a specific link between BPD features and hypermentalizing in adolescent inpatients. Both dimensionally-assessed and categorically-assessed BPD showed significant and non-negligible associations with hypermentalizing. The overall performance on the MASC task significantly discriminated BPD adolescents from Italian community-dwelling adolescents.

**Conclusions:**

Our findings supported the hypothesis that specific deficits in mentalization–namely, hypermentalizing–may play a crucial role in the developmental pathway leading to emerging BPD in adolescence.

Borderline personality disorder (BPD) represents a severe PD that is characterized by an impairing and pervasive dysregulation of affects, self-image, interpersonal relationships, and behavior [1]. BPD is associated with heightened risk for a number of self-destructive behaviors, [2, 3], and it may lead to psychiatric hospitalization [3].

Doubts have been raised as to whether BPD appears de novo at the time an individual turns eighteen [4, 5], stressing the need for early BPD assessment and diagnosis. Early detection of emerging BPD is central also to implement early intervention programs designed to prevent the clinical and social burden that is often associated with BPD in adults [5, 6].

Extant literature indicates that BPD may be reliably assessed in adolescence with validity data that were similar to those observed in adult populations [7]. Epidemiological data suggest that BPD prevalence among general population adolescents (range: 0.9–3.2%) is in close agreement with the adult prevalence.

The development of BPD has been linked to both genetic and environmental factors, supporting the biosocial model [8]. Dysfunctions in mentalizing–i.e., the mental process by which an individual implicitly and explicitly interprets the actions of himself/herself and others as meaningful based on intentional mental states [9]–have been proposed as developmentally-based foundations of BPD subjects’ difficulties in interpersonal relationships [10] and emotion regulation [11].

Using the Movie for the Assessment of Social Cognition [MASC; [12]], a computer-administered laboratory task to assess mentalizing, Sharp and colleagues [11] demonstrated in a sample of 111 U.S. adolescent inpatients that BPD features were selectively associated with hypermentalizing, i.e., with mentalizing errors occurring through the overinterpretation or overattribution of intentions or mental states to others [13], suggesting that mentalization could be an important early target for intervention for influencing the developmental trajectory of BPD. This work also suggested that hypermentalizing may represent an important marker to distinguish emerging BPD from adolescent turmoil. In a number of studies, Sharp and colleagues [14, 15] replicated in subsequent US adolescent inpatient samples the association between hypermentalizing and BPD and showed that hypermentalizing may exert a mediating role in the relationship between trauma and aggression [16]; however, as yet, whether hypermentalizing distinguishes BPD from healthy adolescents remains untested.

Starting from these considerations we aimed at testing if both dimensionally-assessed and categorically-diagnosed BPD was selectively associated with hypermentalizing errors on the MASC task in a sample of Italian adolescent inpatients. In particular, we hypothesized that a) the BPD dimensional score on the Structured Clinical Interview for *DSM-5* Personality Disorders [SCID-5-PD; [17]] was positively and significantly associated with the number of hypermentalizing errors on the MASC task, while showing no significant association with the other MASC dimensions; b) adolescents who met criteria for a *DSM-5* BPD diagnosis showed a significantly higher average number of hypermentalizing errors than subjects with no BPD diagnosis; and c) adolescents who met criteria for a *DSM-5* BPD diagnosis scored significantly higher on the MASC task than normative Italian adolescents. The present study represents the first independent replication of Sharp and colleagues’ [11] findings, particularly in a different cultural context, and extends prior studies by including a healthy adolescent comparison group [18].

## Method

### Participants

The inpatients sample was composed of 58 Italian adolescents who were consecutively admitted to the Adolescent Psychiatry Unit of the Child Psychiatry Department of the “Sapienza” University of Rome, Italy. All participants received a clinical diagnosis of personality pathology. To participate in the study participants had to sign a written informed consent; participants of minor age had to express their assent while their parents had to sign informed consent. IRB approval for the study was obtained. If pervasive developmental disorders, psychosis and intellectual disability were noted by clinicians during initial assessment, participants were not included in the study. Participants’ mean age was 15.25 years, *SD* = 1.34; 34 (58.6%) participants were female. Based on the diagnoses of the clinicians who were following them in treatment, 12(20.7%) participants met criteria for at least one *DSM-5* non-PD mental disorder diagnosis; mood disorders (*n* = 7, 12.1%) were the most frequently diagnosed *DSM-5* non-PD mental disorders.

The MASC normative sample [18] was composed of 373 adolescents attending a public high school in Italy; 238 participants (63.8%) were female; mean age was 17.13 years, *SD* = 1.35 years. A detailed description of the sample is provided in Fossati and colleagues’ [18] validation study.

### Measures

All participants were administered the official Italian translations of the MASC [18] and SCID-5-PD [19, 20]. The SCID-5-PD [17] is a semi-structured interview specifically designed to assess the *DSM-5* Section II PDs that provides both dimensional (i.e., the sum of sub-clinical and clinical scores) and categorical diagnoses. In the present study, interrater reliability (*n* = 20, pairwise interview design) was excellent for both dimensional, random effect one-way ANOVA intraclass *r* = .96, *p* < .001, and categorical, *κ* = 1.00, BPD diagnoses.

The MASC task is a laboratory measure that was designed to assess mentalizing. In the present study, Cronbach *α*s were .76, .78, .91 and .94 for MASC overall performance, and hypermentalizing, hypomentalizing, and no mentalizing scales, respectively.

## Results and discussion

According to SCID-5-PD, 20 (34.5%) adolescents received a *DSM-5* Section II BPD diagnosis; the average SCID-5-PD dimensional rating for BPD (range:0–18) was 7.33, *SD* = 4.86. The small sample size suggested to rely on nonparametric statistics for all comparisons, with the exceptions of the comparisons with normative adolescents. Mann-Whitney *U* test (*U*) was used for median scores comparison; Spearman *r* (*r*_*S*_) was used as a measure of association between continuous variables.

Eighty-five percent (*n* = 17) of the BPD adolescents were female, whereas 44.7% (*n* = 17) of the non-BPD adolescents were female, *χ*^*2*^(1) = 8.76, *p* < .01, *φ* = .39; no significant difference was observed between BPD and non-BPD adolescents on age, *U* = 308.0, *p* > .30, and years of education, *U* = 303.50, *p* > .60.

Among adolescent inpatients, MASC scale scores were not significantly associated with participants’ gender, min. *U* = 337.50 (hypermentalzing), max. *U* = 400.50 (number of correct answers), all *p*s > .20, and years of educations, min. *r*_*S *_(no mentalizing) = −.14, max. *r*_*S *_(hypomentalizing) = .14, all *p*s > .30. MASC hypomentalizing score was significantly associated with participants’ age, *r*_*S*_ = .30, *p* < .05, whereas none of the other MASC scale scores correlated significantly with adolescents’ age with *r*_*S*_ values ranging from −.11 (number of correct answers) to .13 (no mentalizing), all *p*s > .30.

### Associations between MASC scores and BPD ratings

BPD dimensional ratings correlated significantly with the number of MASC hypermentalizing errors, *r*_*S*_ = .30, *p* < .05, while showing no significant association with MASC hypomentalizing errors, *r*_*S*_ = −.26, *p* > .05, no mentalizing errors, *r*_*S*_ = .01, *p* > .50, and number of correct answers, *r*_*S*_ = −.19, *p* > .15. Thus, only hypermentalizing, at least as it is operationalized in the MASC, was significantly associated with both dimensionally-scored and categorically-assessed *DSM-5* BPD in adolescence. Interestingly, the association between dimensionally-scored BPD and hypermentalizing errors on the MASC was not significantly different between male adolescents and female adolescents, *z* = 1.12, *p* > .20. The association between BPD and hypermentalizing is consistent with Sharp and Fonagy’s [21] hypothesis of epistemic hypervigilance as a core feature of personality pathology in adolescence. Interestingly, data on adult BPD participants indicate an association with hypo−/no mentalizing [e.g., [9, 18]] rather than with hypermentalizing, thus indicating that mentalization deficits in BPD may change over time.

### Group comparisons

The MASC scale descriptive statistics and group differences between BPD participants, and non-BPD participants and 373 community-dwelling adolescents (F = 238, 63.8%; mean age = 17.13 years, *SD* = 1.35 years) who took part in the validation study of the Italian translation of the MASC (Fossati et al., 2017) are summarized in Table 1. Our findings showed that hypermentalizing significantly and substantially discriminated BPD adolescents from non-BPD adolescents in the inpatient sample, as well as BPD adolescents from community-dwelling adolescents. In the latter comparison also the general performance on the MASC significantly differentiated BPD adolescents from the normative sample. In our study, 83.9% of the community-dwelling adolescents were below the average hypermentalizing score on the MASC of the BPD adolescent group, whereas 80.9% of the non-BPD adolescent inpatients showed MASC hypermentalizing scores that fell below the mean hypermentalizing value of BPD adolescent inpatients (a rank point biserial *r* value of .40 corresponds to a Cohen’s *d* value of 0.873). Finally, it should be observed that roughly 77.4% of our BPD adolescents was below the mean value of the community-dwelling adolescents for the number of correct responses on the MASC, suggesting that even general impairment in mentalistic abilities may be considered in recognizing adolescents with emerging BPD features among community-dwelling adolescents.

**Table 1:** Movie for the Assessment of Social Cognition scale scores: Descriptive statistics and group comparisons between BPD adolescents, and non-BPD adolescents and community-dwelling adolescents, respectively

	Adolescent Inpatients (*n* = 58)		BPD Adolescent Inpatients (*n* = 20)		Non-BPD Adolescent Inpatients (*n* = 38)				Community-Dwelling Adolescents^1^ (*N* = 373)			
MASC Scales	*M*	*SD*	*M*	*SD*	*M*	*SD*	*U*	rank *r*_*pb*_	*M*	*SD*	*t*(391)	*d*
Hypermentalizing	11.26	4.03	13.10	4.27	10.29	3.59	229.50^*^	.40	10.01	3.19	4.31^***^	0.99
Hypomentalizing	4.83	2.62	4.15	2.11	5.18	2.81	282.00	.26	3.94	2.26	0.41	0.09
No Mentalizing	3.36	2.55	2.95	2.52	3.58	2.56	314.50	.17	3.02	2.27	−0.14	−0.03
Total number of Correct Responses	25.59	5.17	24.80	4.99	26.00	5.28	302.50	.20	28.08	4.47	−3.27^**^	−0.75

*Note*. 1: From Fossati and colleagues’ (2017) validation study of the MASC in Italy, *MASC* Movie for the Assessment of Social Cognition, *BPD* Borderline personality disorder; rank *r*_*pb*_: rank point-biserial r coefficient; *d*: Cohen’s *d* coefficient

*p < .05; **p < .01; ***p < .001

## Conclusions

To our knowledge, our study represents the first replication of Sharp and colleagues’ [11] findings in an independent sample of adolescent inpatients from a different culture. Our findings supported the hypothesis that specific deficits in mentalization – namely, hypermentalizing – may play a crucial role in the developmental pathway leading to emerging BPD in adolescence [e.g., [11, 15]]. Specifically, the effect size estimates indicated that clinicians should not overlook the relevance of hypermentalizing for BPD assessment and treatment in adolescents. Of course, our conclusions should be considered in the light of several limitations. The sample size is small, and the sample included only inpatient participants. We relied only on the MASC task to evaluate mentalizing, as well as only on the SCID-5-PD to assess BPD. These limitations stress the need for further studies on this topic.

## Abbreviations

BPD: Borderline personality disorder
*DSM-5*: Diagnostic and statistical manual-5th edition
MASC: Movie for the assessement of social cognition
PD: Personality disorder
SCID-5-PD: Structured Clinical Interview for *DSM-5* Personality Disorders
